# Proteomic analysis of serum extracellular vesicles from biliary tract infection patients to identify novel biomarkers

**DOI:** 10.1038/s41598-024-56036-y

**Published:** 2024-03-08

**Authors:** Chih-Jung Chang, Yung-Ning Huang, Yang-Bor Lu, Yi Zhang, Ping-Hua Wu, Jian-Shan Huang, Wei Yang, Tung-Ying Chiang, Hui-Shan Hsieh, Wen-Hung Chung, Yu-Chieh Weng

**Affiliations:** 1 School of Medicine and Medical Research Center, Xiamen Chang Gung Hospital Hua Qiao University, Quanzhou and Xiamen, Fujian China; 2 Department of Digestive Disease, Xiamen Chang Gung Hospital Hua Qiao University, Xiamen, Fujian China; 3 Hepatobiliary and Pancreatic Unit, Xiamen Chang Gung Hospital Hua Qiao University, Xiamen, Fujian China; 4 Department of Emergency Clinic, Xiamen Chang Gung Hospital Hua Qiao University, Xiamen, Fujian China; 5 Department of Otolaryngology-Head and Neck Surgery, Sleep Center, Xiamen Chang Gung Hospital Hua Qiao University, Xiamen, Fujian China; 6 Drug Hypersensitivity Clinical and Research Center, Department of Dermatology, Chang Gung Memorial Hospital, Linkou, Taoyuan, Taipei and Keelung, Taiwan; 7 Department of Dermatology, Xiamen Chang Gung Hospital Hua Qiao University, Xiamen, Fujian China

**Keywords:** Predictive markers, Proteomics

## Abstract

Biliary tract infection (BTI), a commonly occurring abdominal disease, despite being extensively studied for its initiation and underlying mechanisms, continues to pose a challenge in the quest for identifying specific diagnostic biomarkers. Extracellular vesicles (EVs), which emanate from diverse cell types, serve as minute biological entities that mirror unique physiological or pathological conditions. Despite their potential, there has been a relatively restricted exploration of EV-oriented methodologies for diagnosing BTI. To uncover potent protein biomarkers for BTI patients, we applied a label-free quantitative proteomic method known for its unbiased and high-throughput nature. Furthermore, 192 differentially expressed proteins surfaced within EVs isolated from individuals afflicted with BTI. Subsequent GO and KEGG analyses pinpointed Carcinoembryonic antigen-related cell adhesion molecule 1 (CEACAM1) and Crumbs homolog 3 (CRB3) as noteworthy biomarkers. Validation via data analysis of plasma-derived EV samples confirmed their specificity to BTI. Our study leveraged an unbiased proteomic tool to unveil CEACAM1 and CRB3 as promising protein biomarkers in serum EVs, presenting potential avenues for the advancement of diagnostic systems for BTI detection.

## Introduction

Acute cholecystitis and acute cholangitis are both encompassed within the category of biliary tract infection (BTI), a life-threatening condition arising from the synergistic interplay of infection and the obstruction of small interlobular bile ducts. BTI stands as the primary cause of bacteremia^1^. The infection of the biliary tract manifests as inflammation of the gall bladder wall and bile ducts, culminating in the diagnosis of acute or chronic cholangitis^2^. Bacteremia, a prevalent instigating factor for BTI, is closely linked to heightened morbidity and mortality, particularly in older patients. Despite notable advancements in the clinical outcomes of patients with acute BTI over the past decades, with mortality rates decreasing from 50 to 10%, the mortality remains elevated for severe BTI cases in patients lacking systematic treatment^3,4^.

Extracellular vesicles (EVs) represent bilayer-delimited particles released by virtually all cell types^5,6^. These vesicles facilitate the transport of diverse cargoes, encompassing lipids, nucleic acids, and proteins, thereby enabling intercellular exchange of these components^6,7^. Presently, the scientific and clinical interest in these double-layer phospholipid membrane vesicles is burgeoning due to their pivotal roles in intercellular communication, immune responses, cellular homeostasis, and pathological progressions^8–13^. Furthermore, accumulating evidence highlights the significant involvement of EV secretion in various human gastrointestinal diseases, such as gastric cancers^14^.Given that EVs are released into bodily fluids, such as blood and urine, by cells of diverse origins, the exploration of EV-derived specific cargos, notably enriched in RNAs and proteins, is underway as potential biomarkers for various diseases. Nevertheless, the functional implications and predictive values of EVs in BTI remain underexplored. The identification of key protein biomarkers from EVs in serum, collected non-invasively, holds profound significance for diagnosing patients with BTI.

Proteomic profiling technologies enable comprehensive protein annotations in a high-throughput and unbiased manner. These methods process proteins from various biological samples, including cells, tissues, or serum. Apart from their role in identifying crucial proteins with altered levels or post-translational modifications during specific biological processes, proteomics-based investigations are extensively employed for screening pivotal biomarkers for diagnosing and prognosticating different diseases^15^. For instance, Ming et al. conducted quantitative label-free proteomic analysis on serum protein samples from choledochal cysts patients, identifying 47 differentially expressed proteins potentially useful as biomarkers for clinical diagnosis^16^. Despite the widespread use of peripheral blood in proteomic analyses, the intricate composition of serum, such as albumin and immunoglobulins, might impede accurate biomarker detection using proteomic approaches^17^. Consequently, serum-derived EVs are emerging as a preferred source for developing proteomics-based biomarker identification, circumventing challenges associated with serum complexity.

While certain proteins in EVs have shown promise as biomarkers for biliary tract-related diseases like perihilar cholangiocarcinoma^18^, the identification of effective protein biomarkers, particularly through high-throughput methods, for diagnosing BTI remains understudied. In our pursuit of screening protein biomarkers in an unbiased manner, we conducted label-free quantitative proteomic analysis on EVs isolated from the serum of healthy control donors and BTI patients using liquid chromatography-mass spectrometry. Our findings revealed 62 proteins uniquely enriched in EVs derived from BTI patients compared to those from healthy controls. Moreover, we identified 192 differentially expressed proteins, further subjecting them to systematic investigation through bioinformatic analysis to discern the biological processes and pathways they are associated with. Carcinoembryonic antigen-related cell adhesion molecule 1 (CEACAM1) and Crumbs homolog 3 (CRB3) were notably prominent in serum EVs of BTI patients and were subsequently validated. Collectively, our results offer valuable protein candidates worthy of clinical investigation and potential development into protein biomarkers for diagnosing BTI in patients.

## Results

### Extraction and characterization of EVs

**Figure 1:**
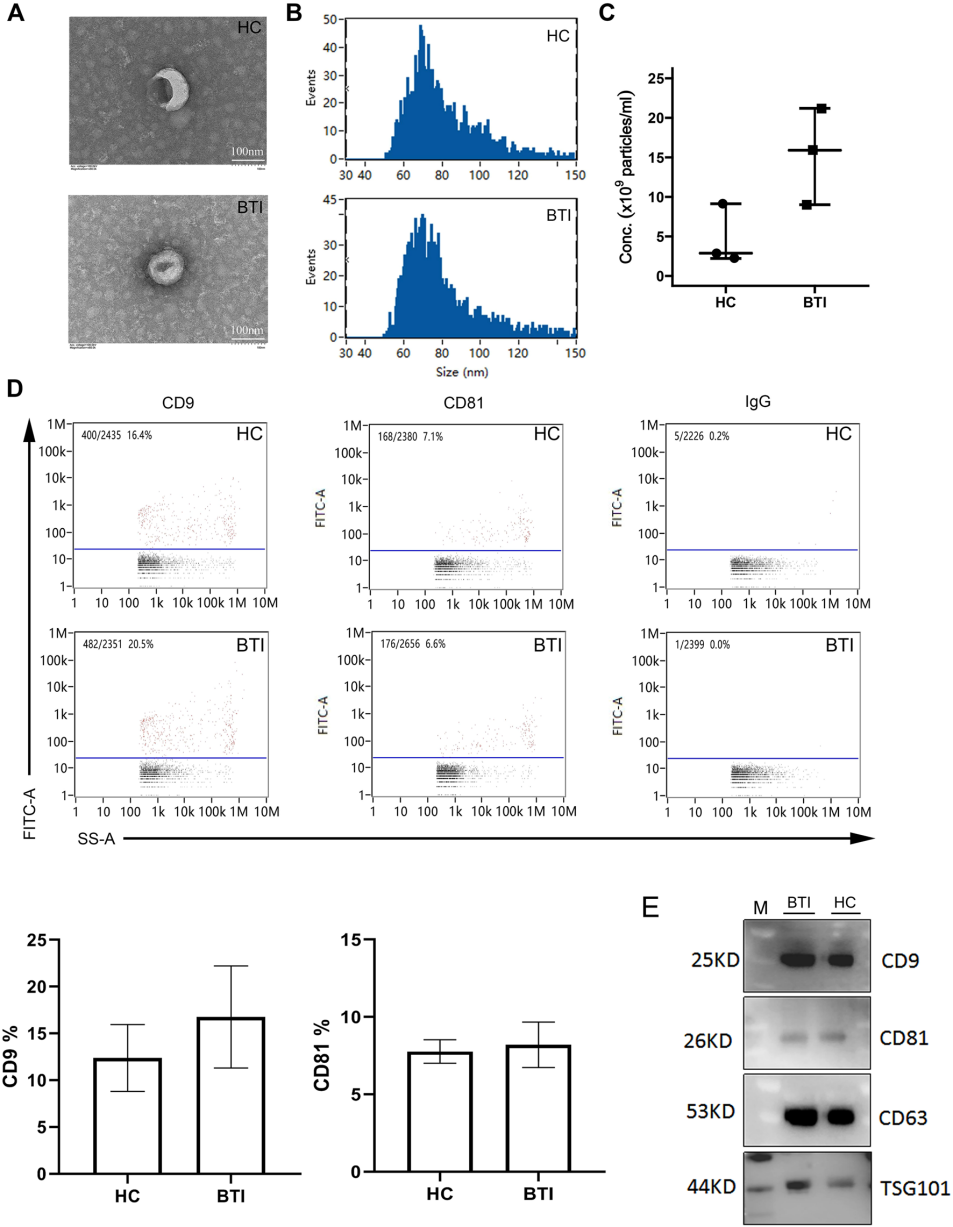
Characterization of EVs (extracellular vesicles) from HC (healthy control) and BTI (biliary tract infection) samples. (**A**) Representative images of EVs, which were captured by TEM (Transmission Electron Microscope), from HC or BTI samples (bar = 100 nm). (**B**, **C**, **D**) Plot showing the sizes (**B**) or concentrations (Conc.; **C**) and cell surface expression of CD9/CD81/IgG (**D**) of EVs which were measured by the NFCM (Nano-Flow Cytometry Measurement). (**E**) CD9, CD81, CD63 and TSG101 were detectable by Western blot (WB) analysis.

To elucidate the differential protein expression in EVs derived from healthy donors (HC, n = 3) and patients diagnosed with biliary tract infection (BTI, n = 3), platelet-free plasma was collected following standard procedures from the serum of HC and BTI patients. Subsequently, plasma EVs were isolated and characterized using transmission electron microscope (TEM). As shown in Fig. 1A, cup-shaped vesicles observed in representative samples from an HC and a BTI patient, affirming the successful extraction and structural integrity of plasma EVs. Furthermore, a nano-flow cytometer (NFCM) was employed to quantify the size and concentration of the isolated EVs. The diameter of EVs from HC or BTI patients ranged from 50 to 150 nm, with comparable average sizes of 79.23 ± 1.63 nm and 75.31 ± 5.28 nm, respectively (Fig. 1B). Additionally, the concentrations of EVs in HC and BTI patients were determined as 4.74 × 109 ± 3.82 × 109 p/mL and 15.4 × 109 ± 6.11 × 109 p/mL, respectively, with no significant difference observed (*p* = 0.0625), despite a higher concentration of EVs from BTI patients compared to HC (Fig. 1C). Moreover, the presence of EV surface proteins CD9 and CD81^19,20^ was confirmed in all six samples using IgG as a control, providing molecular characterization of EVs (Fig. 1D). These findings collectively indicate the successful extraction of high-quality EVs from both HC and BTI patients. Additionally, the detection of specific EV markers, CD9, CD63, CD81, and TSG101, was verified through western blot analysis, confirming the presence of exosomal markers (Fig. 1E).

### Proteomic analysis of EVs

**Figure 2:**
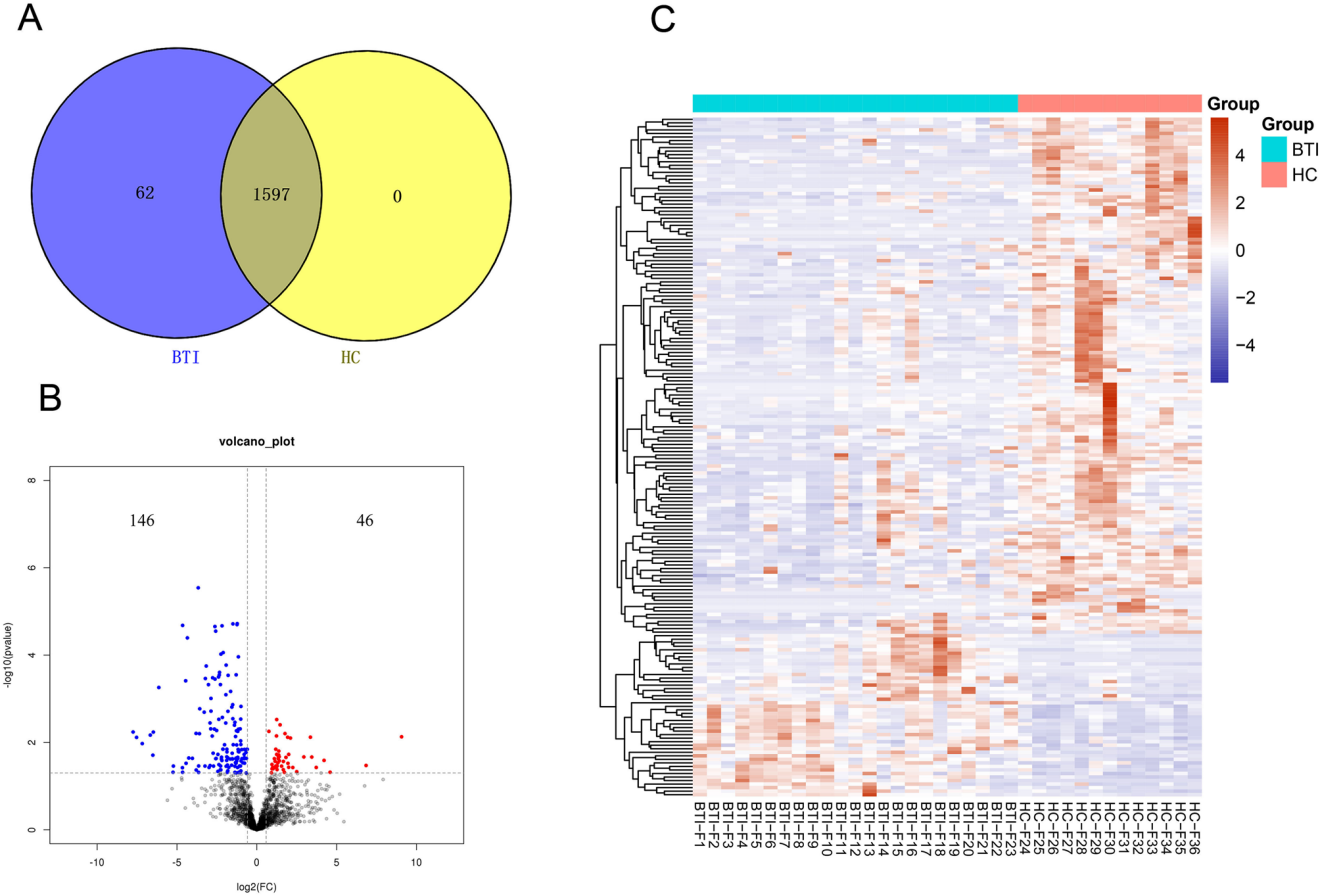
The differentially expressed proteins in EVs. (**A**) Venn diagram. (**B**) Proteins that were significantly upregulated or downregulated in BTI EV samples were demonstrated as red and blue, respectively. (**C**) Hierarchical clustering heat map indicating the differentially expressed proteins in BTI EV samples, with a tree on the left side indicating the clustering relationship of proteins.And heat maps, were created by heatmap3 package in R.

Liquid chromatography–mass spectrometry (LC–MS/MS) was conducted on EVs isolated from both HC and BTI patients to systematically map their protein profiles. The LC–MS/MS data revealed an enrichment of a larger quantity of proteins from both groups (HC = 23, BTI = 13). Specifically, 1659 proteins were identified as common in BTI patient samples, while 1597 proteins were commonly expressed in both HC and BTI samples (Fig. 2). This suggests a relatively similar protein profiling between EVs obtained from HC and BTI patients. However, 62 proteins were uniquely present in the BTI samples compared to those from HC (Fig. 2A, supplementary Table 1). Additionally, a volcano plot unveiled 192 proteins expressed differentially between the two groups (Fig. 2B, supplementary Table 2). Among these proteins, 46 exhibited upregulation, while 146 showed downregulation in the BTI samples (Fold change > 1.5, *p* < 0.05, supplementary Table 1. Tables 1 and 2 respectively showcase the top 15 upregulated and downregulated proteins. In the top 15 up-regulated and down-regulated proteins of differentially expressed proteins, 18 (60%) were archived in the publicly available extracellular vesicle databases ExoCarta, suggesting that the majority of proteins differentially expressed in the current study are well documented in the extracellular fraction, proving the value of this dataset. Finally, a heatmap visualized the clustering of differentially expressed proteins (Fig. 2C).

**Table 1:** The top 15 upregulated proteins of differential expressed proteins.

Protein IDs	Protein name	Gene name	Fold Change	*p*-value	Related pathology*
P13688	Carcinoembryonic antigen-related cell adhesion molecule 1	CEACAM1	535.694	2.817E−04	Colorectal cancer; Melanoma
Q9BUF7	Protein crumbs homolog 3	CRB3	114.878	2.941E−03	Colorectal cancer
Q86VV4	Ran-binding protein 3-like	RANBP3L	24.100	5.272E−03	NA
P08473	Neprilysin	MME	18.159	1.969E−03	Bladder cancer; Melanoma
P15144	Aminopeptidase N	ANPEP	13.187	3.760E−03	Colorectal cancer; Melanoma; Mesenchymal stem; Neuroblastoma; Ovarian cancer
P08319	All-trans-retinol dehydrogenase [NAD( +)] ADH4	ADH4	10.863	1.327E−03	NA
Q16651	Prostasin	PRSS8	10.238	3.016E−04	Colorectal cancer; Ovarian cancer
P21589	5'-nucleotidase	NT5E	7.701	1.294E−03	Bladder cancer; Colorectal cancer; Hepatocellular carcinoma; Melanoma; Mesenchymal stem
A0M8Q6	Immunoglobulin lambda constant 7	IGLC7	5.653	5.063E−03	NA
Q8IZ83	Aldehyde dehydrogenase family 16 member A1	ALDH16A1	4.765	3.635E−03	Colorectal cancer; Ovarian cancer
O75821	Eukaryotic translation initiation factor 3 subunit G	EIF3G	4.304	3.254E−04	Hepatocellular carcinoma; Ovarian cancer
P02765	Alpha-2-HS-glycoprotein	AHSG	3.987	1.052E−03	Colorectal cancer; Hepatocellular carcinoma; Hepatocytes; Melanoma; Mesenchymal stem; Neuroblastoma; Prostate cancer
Q6UWD8	Transmembrane protein C16orf54	C16orf54	3.986	3.570E−03	NA
Q01628	Interferon-induced transmembrane protein 3	IFITM3	3.862	2.537E−03	Colorectal cancer; Hepatocellular carcinoma; Ovarian cancer
P01817	Immunoglobulin heavy variable 2–5	IGHV2-5	3.814	2.981E−04	NA

*If a protein has been previously identified in exosomes, information on the related pathology is provided. "NA" signifies that the protein has not been identified in exosomes related to any specific pathology.

**Table 2:** The top 15 down-regulated proteins of differential expressed proteins.

Protein IDs	Protein name	Gene name	Fold Change	*p*-value	Related pathology*
O75947	ATP synthase subunit d, mitochondrial	ATP5PD	− 214.747	1.912E−04	NA
P21912	Succinate dehydrogenase [ubiquinone] iron-sulfur subunit, mitochondrial	SDHB	− 184.880	3.092E−04	NA
P47985	Cytochrome b-c1 complex subunit Rieske, mitochondria	UQCRFS1	−144.406	4.480E−04	NA
Q9P2N5	RNA-binding protein 27	RBM27	−101.810	2.454E−04	NA
Q15067	Peroxisomal acyl-coenzyme A oxidase 1	ACOX1	−91.101	1.144E−03	NA
Q14966	Zinc finger protein 638	ZNF638	−89.267	1.956E−04	Melanoma
O75443	Alpha-tectorin	PRSS8	−70.363	8.644E−06	Colorectal cancer; Ovarian cancer;
Q53GQ0	Very-long-chain 3-oxoacyl-CoA reductase	HSD17B12	−37.981	5.306E−03	Ovarian cancer
O15551	Claudin-3	CLDN3	−37.415	3.183E−03	Colorectal cancer; Hepatocellular carcinoma; Ovarian cancer; Prostate cancer; Squamous carcinoma
Q9Y2I8	WD repeat-containing protein 37	WDR37	−25.286	3.837E−03	Hepatocellular carcinoma
Q99424	Peroxisomal acyl-coenzyme A oxidase 2	ACOX2	−25.089	5.254E−03	NA
Q9NZT1	Calmodulin-like protein 5	CALML5	−25.064	8.804E−08	Liver cancer
Q8IUZ5	5-phosphohydroxy-L-lysine phospho-lyase	PHYKPL	−21.950	5.377E−06	NA
Q9H3N1	Thioredoxin-related transmembrane protein 1	TMX1	−21.585	2.515E−03	Squamous carcinoma
P09769	Tyrosine-protein kinase Fgr	FGR	−20.379	2.187E−07	Hepatocytes; Neuroblastoma; Squamous carcinoma

*If a protein has been previously identified in exosomes, information on the related pathology is provided. "NA" signifies that the protein has not been identified in exosomes related to any specific pathology.

### GO and KEGG analysis of differentially expressed proteins

**Figure 3:**
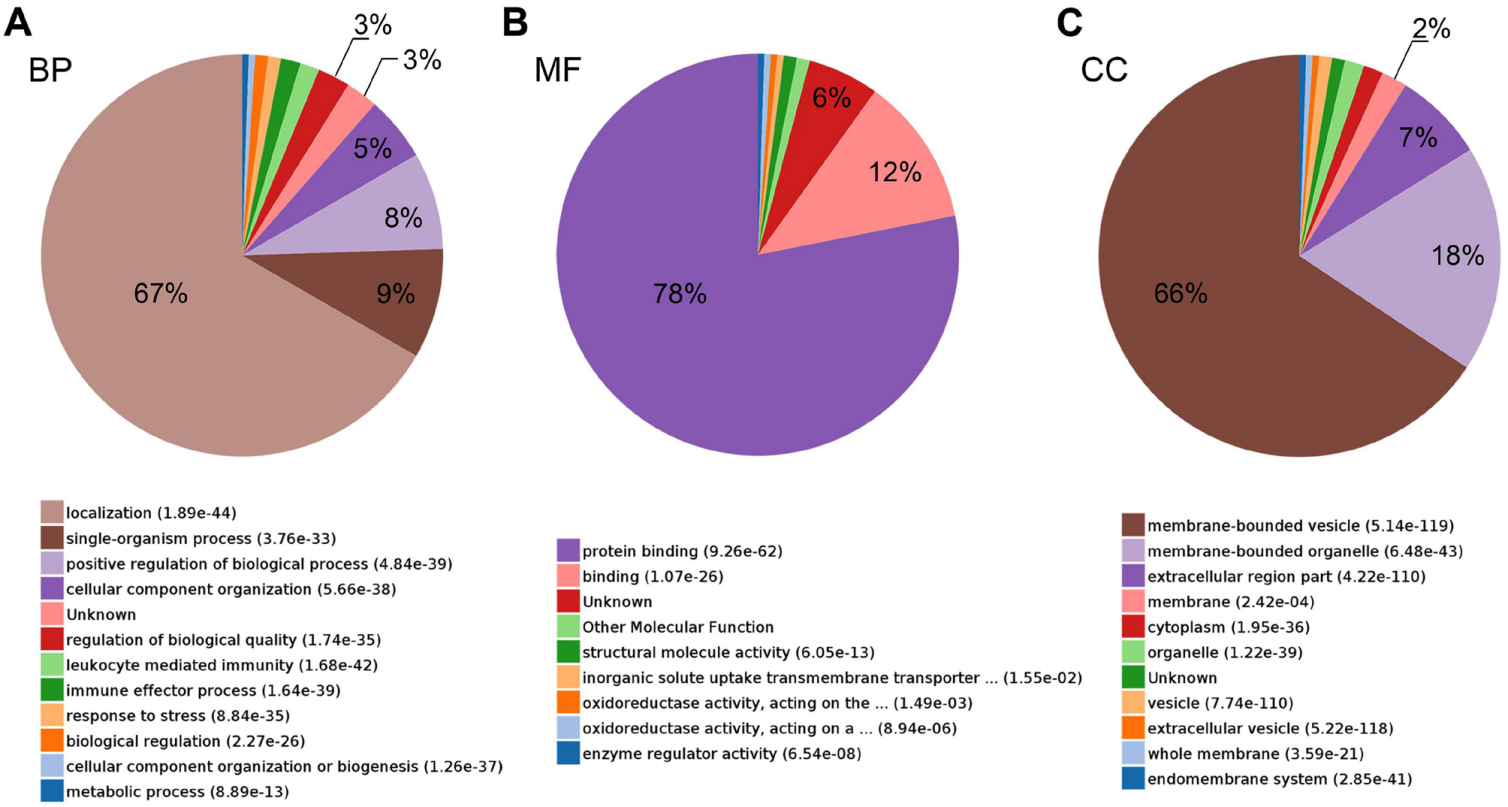
GO analysis of the differentially expressed proteins in EVs. (**A**, **B**, **C**) The biological process (BP; **A**), molecular function (MF; **B**) and cellular component (CC; **C**) of the differentially expressed proteins in EVs were determined.

**Figure 4:**
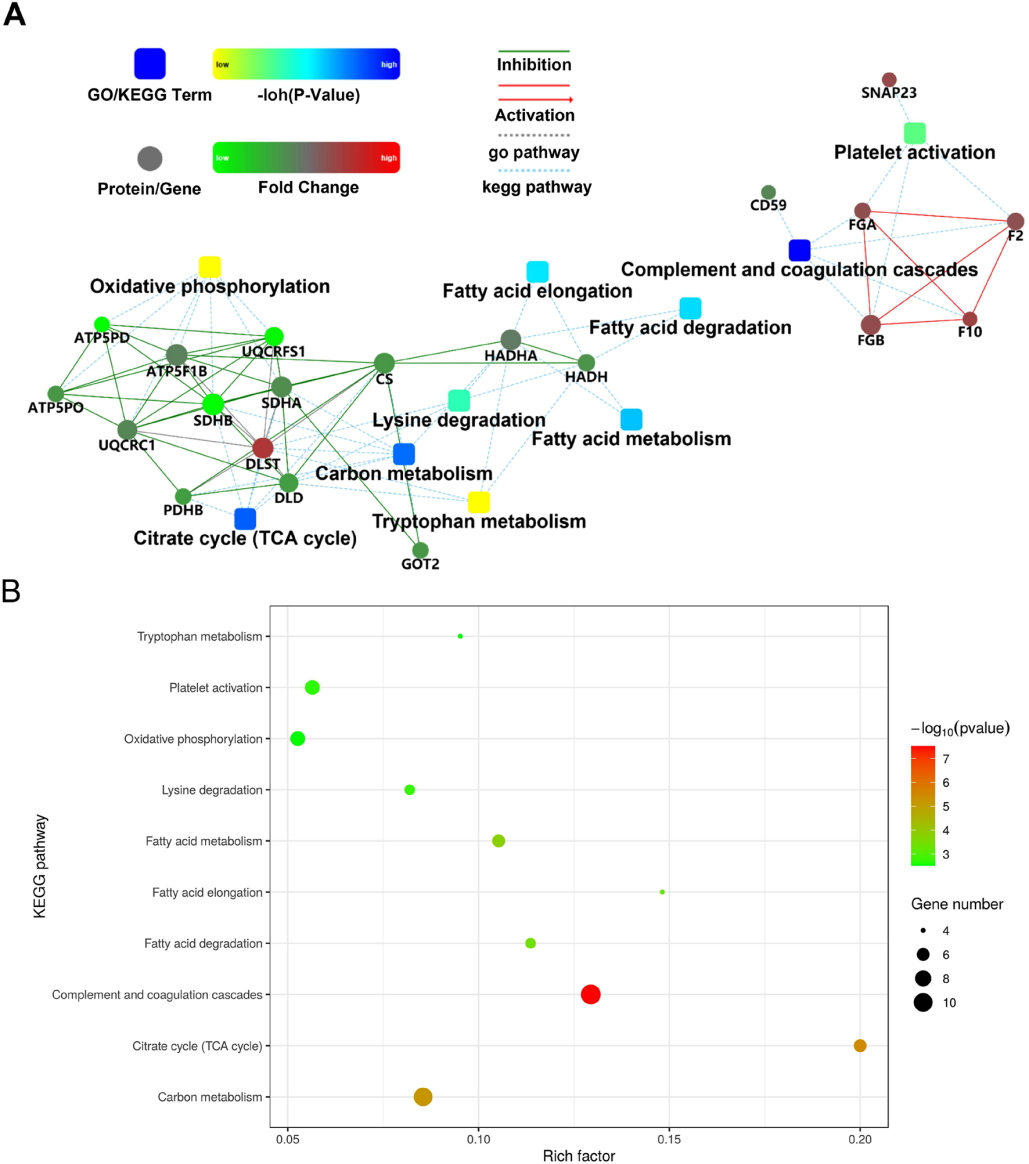
PPI network and KEGG pathway analysis of the differentially expressed proteins in EVs. (**A**) PPI networks of the differentially expressed proteins in EVs were mapped by the STRING software. (**B**) Bubble map showing the top 10 related KEGG pathways. Permission has been obtained from Kanehisa laboratories for using KEGG pathway database^46^.

Despite the detection of a variety of differentially expressed proteins, the biological roles of these proteins remain elusive. Consequently, GO analysis was conducted to delve deeper into the functional annotation of the 192 differentially expressed proteins. The data revealed that a majority of these proteins (67%) were implicated in cell localization, while 8% and 9% respectively exhibited potential involvement in single-organism processes and positive regulation of biological processes (Fig. 3A). Molecular function analysis of these protein candidates indicated their predominant roles in protein binding (78%) and binding activities (12%) (Fig. 3B). Additionally, 66% of these proteins were associated with membrane-bounded vesicles, with 18% and 7% speculated to localize in membrane-bound organelles and extracellular regions, respectively (Fig. 3C). Subsequently, STRING analysis was employed to further explore the protein–protein interactions (PPI) among the protein candidates localized in membrane-bounded vesicles. Notably, these proteins formed an enriched PPI network, exhibiting correlations with KEGG pathways such as the citrate cycle, fatty acid elongation, complement and coagulation cascades, fatty acid degradation, and fatty acid metabolism (Fig. 4A and B).

### Biomarker candidates were altered and validated in serum EVs samples patients with BTI patients

**Figure 5:**
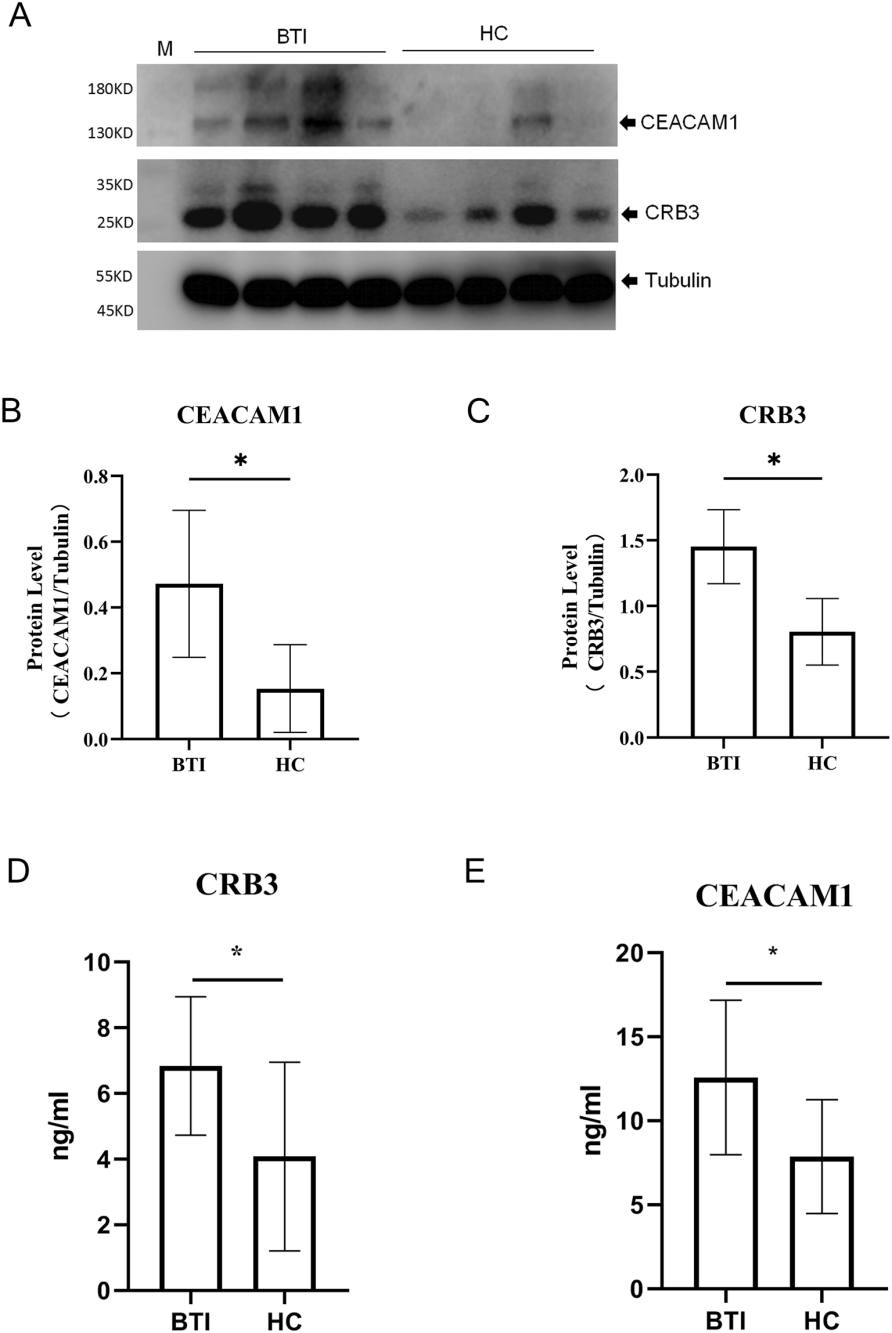
Validation of CEACAM1 and CRB3 expression in serum EVs. (**A**) CEACAM1 and CRB3 were detectable by Western blot (WB) analysis (N total = 8). (**B**) Densitometric quantification of CEACAM1. (**C**) Densitometric quantification of CRB3. (**D**) CRB3 was detectable by ELISA analysis. (**E**) CEACAM1 was detectable by ELISA analysis.* p < 0.05.

In Table 1, CEACAM1 and CRB3 exhibited significantly higher fold-increases in BTI patients compared to healthy individuals. To validate the alterations observed in CEACAM1 and CRB3, additional BTI patients and healthy individuals (n = 10) were recruited to assess the protein levels in serum-derived EVs. Western blot analysis was conducted on serum EVs samples using antibodies specific to these proteins (n = 4). Twenty micrograms of serum EVs proteins from four patients and four healthy individuals were subjected to analysis using anti-CEACAM1 and anti-CRB3 antibodies, enabling the detection of the separated proteins on membranes (Fig. 5A). The western blot densities of CEACAM1 and CRB3 were quantified, showcasing increased expression in BTI patients (Fig. 5B and C), consistent with the findings derived from the EV proteomic analysis comparing patients and healthy individuals. For further validation, the expression of CEACAM1 and CRB3 proteins in the serum of BTI patients and healthy individuals was assessed using ELISA experiments (n = 10), corroborating the results obtained from the Western blot experiments (Fig. 5D and E).

## Discussion

Biliary tract infection, a prevalent cholestatic liver disease, primarily stems from bacterial infections, inciting inflammation that can potentially progress to cirrhosis and liver failure^21^. The condition's high morbidity and mortality rates, coupled with delayed diagnoses, have underscored its emergent status as a life-threatening ailment in recent years^22^. A comprehensive Asian study led by Gomi et al.^23^ demonstrated an escalating 30-day all-cause mortality rate, with a 2.4% increase in low-severity acute cholangitis and an 8.4% rise in high-severity cases. Consequently, substantial attention has been directed toward exploring potential biomarkers that could furnish clinical reference data for diagnosing and treating BTI patients. Despite certain proteins, such as site-specific glycan classifiers, exhibiting diagnostic promise for BTI patients^24^, the quest for effective diagnostic biomarkers persists to enable a more precise diagnosis leveraging multiple biomarkers for BTI patients.

For a considerable period, cell-released EVs were deemed as biological byproducts devoid of notable physiological significance. This perception was challenged upon the revelation that EVs encapsulate a substantial array of bioactive molecules, comprising DNA, RNA, and proteins^25^. To pave the way for this burgeoning field, methodologies for isolating EVs with integrity, characterizing and labeling EVs, and functionally probing their attributes have rapidly devolved^26^. EVs have emerged as pivotal facilitators of intercellular communication, engaging in diverse functions by transporting cargoes between cells^27^. Notably, EV-focused approaches for diagnosing and prognosticating diseases have shown substantial clinical promise. For instance, EV-derived miRNAs have been instrumental in diagnosing various cancer types^28^. However, the inherent instability of RNAs has limited the widespread application of EV-derived miRNAs for diagnostic purposes. Therefore, the exploration of protein biomarkers, which possess greater biological stability, assumes paramount significance.

Due to the absence of established protein biomarkers for identifying patients with BTI, our study focused on discerning effective biomarkers by comparing the protein profiles of EVs derived from healthy controls and BTI-diagnosed patients. To achieve this, high-purity EVs were isolated from platelet-free plasma obtained from both HC and BTI patients. Notably, NFCM analysis revealed no significant differences in the size and concentration of extracted EVs between normal and BTI samples. Subsequently, label-free quantitative proteomics was employed to delve deeply into the differential expression of proteins. This marks the inaugural application of proteomic analysis on plasma EVs from BTI patients. Analysis revealed 62 proteins exclusively expressed in EV samples from BTI patients, while 1597 proteins were commonly expressed in both HC and BTI samples. Notably, differential expression analysis identified 192 enriched proteins in EVs from BTI patients compared to healthy controls. To gain deeper insights into the processes and pathways associated with these differentially expressed proteins, GO and KEGG analyses were conducted. The citrate cycle emerged as the most pertinent pathway, while pathways related to fatty acid processing, including fatty acid elongation, degradation, and metabolism, were notably enriched. This observation suggests potential dysregulation in essential metabolic processes of biliary tract cells due to infection, evident in the perturbed citrate cycle associated with aerobic glycolysis in BTI patients^29^. Surprisingly, fatty acid processing pathways were also enriched. Given the limited investigation into the role of fatty acids in BTI, further exploration of their biological effects and underlying mechanisms on biliary tract cells holds promise for a deeper understanding of BTI pathophysiology.

Based on the differential expression of proteins identified through quantitative LC–MS/MS analysis, we conducted Western blot measurements (n = 4) of the top increased proteins, CEACAM1 and CRB, and validated using ELISA in a larger sample size (n = 10). CEACAM1, a highly glycosylated cellular adhesion molecule, is part of the carcinoembryonic antigen cell family, and previous research has underscored its involvement in immunoregulation, encompassing functions such as cell adhesion, migration, and intercellular signaling^30,31^. Its expression spans various cell types, including epithelial cells, endothelial cells, activated T cells, and neutrophils^31,32^. CEACAM1 has been implicated in modulating proliferation, function, and cytokine secretion by synergistically regulating the TIM-3 molecule in T cells^33,34^. In activated neutrophils, elevated CEACAM1 rapidly translocates to the cell surface, becoming available in plasma^35^. Studies in patients with ischemic stroke have shown increased plasma CEACAM1, correlating positively with CEACAM1 levels on neutrophils^36^. Similarly, patients with rheumatoid arthritis exhibit higher surface expression of CEACAM1 compared to healthy individuals^37^. Additionally, CEACAM1 has been associated with liver disease^38^, being released into human bile, serving as an indicator for obstructive and inflammatory liver diseases^38^. In the context of BTI, characterized by the infiltration of neutrophils^39^ and T cells^40^ into bile ducts, the accumulation of intracellular CEACAM1 might be packaged into EVs and released in BTI patients. CRB3, a member of the Crumbs protein family, is involved in tight junction formation, thereby maintaining epithelial polarity and homeostasis^41^. Epithelial cells, belonging to the innate immune system, typically harbor Toll-like receptors to recognize pathogens.

EVs originating from epithelial cells are implicated in the activation, migration, and cytokine production of immune cells^42^. Previous studies have delineated the release of EVs from epithelial cells mediated by Toll-like receptor 4 (TLR4)/IKK2 during biliary infection^43^. This mechanism suggests the potential overproduction of EVs from epithelial cells, subsequently released into the circulation. However, investigations regarding protein biomarkers in BTI patients are notably absent. To the best of our knowledge, our study is the pioneering effort in demonstrating the utility of protein biomarkers for diagnosing BTI.

## Conclusion

In summary, our investigation highlights the utility of label-free quantitative proteomics in identifying potential protein candidates within EVs derived from the serum of BTI patients. To our knowledge, this study represents the inaugural exploration of EV biomarkers in BTI diagnosis using unbiased proteomic analysis. Our findings have unveiled a spectrum of differentially expressed proteins, among which CEACAM1 and CRB3 emerge as promising candidates with the potential to serve as valuable biomarkers in the clinical diagnosis of BTI patients. Furthermore, delving into the evaluation and exploration of their molecular mechanisms promises to furnish a deeper comprehension of BTI initiation and progression. These insights hold the potential to significantly advance therapeutic approaches for BTI patients, ultimately contributing to improved clinical outcomes in BTI management.

## Material and methods

### Serum collection

This study encompassed 23 cases of BTI patients and 13 healthy donors recruited from Xiamen Chang Gung Hospital between August 2020 and August 2022. The participant cohort comprised 19 males and 17 females, with an average age of 49.1 years (27–87 years). Serum samples were systematically collected from all individuals. Additionally, 10 each of BTI patients and healthy donors were recruited for further validation, including 9 males and 11 females with an average age of 48.5 years (29–78 years), were randomly selected for further validation. Diagnosis of patients in the BTI group adhered to the Tokyo guidelines 2018^44^. Blood samples were procured in the emergency department to minimize any potential interference from antibiotics. Healthy individuals exhibited no BTI symptoms and had not taken antibiotics within the preceding month. Platelet-free plasma was obtained by subjecting samples to centrifugation twice at the lowest deceleration of 2500 g for 15 min to separate erythrocytes, leukocytes, platelets, and other blood cells. The resulting supernatant, excluding the last 0.5 cm towards the bottom, was meticulously transferred to a clean tube to eliminate residual small platelets and erythrocyte remnants. Uniform procedures were rigorously implemented across all samples in this study. The entire serum collection process strictly adhered to the ethical principles outlined in the Helsinki Declaration and was approved by the Ethics Committee of Xiamen Chang Gung Hospital (NO. CMRPG1G0161). Written informed consent was obtained from all participants involved in this research.

### Extraction of EVs

Serum-derived EVs were isolated using the widely employed ultracentrifugation-based method. Initially, 4 mL blood samples were diluted to 20 mL using pre-chilled phosphate-buffered saline (PBS) to minimize viscosity. Subsequently, the diluted blood samples underwent centrifugation at 2000 g for 30 min at 4 °C to eliminate debris from the serum. The supernatant was then transferred to a fresh tube and subjected to a 45-min spin at 10,000 g (4 °C). Following this, filtration was executed using 0.45 μm syringe filters on the aforementioned samples, and the resulting supernatants were subjected to ultracentrifugation (Optima L-100XP, Beckman) at 100,000 g for 1 h. Subsequently, the EV pellets obtained were resuspended in cold PBS (10 mL) and subjected to another round of ultracentrifugation. Ultimately, the EV pellets were resuspended in 100 μL PBS and stored at  − 80 °C until further investigations were conducted. Notably, freeze–thaw cycles were strictly avoided.

### Sample preparation

The isolated EVs underwent lysis using 1% Rapigest (100 μL/sample, Waters) dissolved in ammonium bicarbonate (50 mM). Post-lysis, the samples were subjected to boiling with dithiothreitol (20 mM, Sigma) for 5 min, followed by alkylation with iodoacetamide (50 mM, Sigma) at room temperature for 30 min. Subsequently, the lysates underwent centrifugation at 20,000 g for 10 min, and the resulting supernatants were filtered using 10 kDa spin filters (Millipore). Sequentially, the samples underwent triple washing with 8 M urea and double washing with ammonium bicarbonate. Each wash cycle involved centrifugation at 13,500 g. Following the washes, trypsin was added at a protein/enzyme ratio of 50:1 for overnight tryptic digestion at 37 °C. The resultant peptides were retrieved from the filter by centrifugation at 14,000 g for 10 min (CP100MX, Hitachi High-Technologies Corporation, Japan). Additionally, the filters underwent two further washes with ammonium bicarbonate to optimize recovery efficiency. Lastly, StageTips (Empore™) were employed for sample desalination, followed by speed vacuum to dry the samples.

### Transmission electron microscope (TEM) assessment

Negative staining TEM was employed to visualize and analyze the morphology of the isolated EVs. In summary, 10 μL of EVs, diluted in 1% uranyl acetate, were meticulously deposited onto a copper grid and allowed to settle for 60 s. Subsequently, excess buffer containing exosomes was removed using filter paper. The EVs adhering to the copper grid were then stained with 2% uranyl acetate for 60 s, following the removal of excess solution with filter paper. The prepared copper grids were air-dried at room temperature for several minutes, and images of the EVs were captured using TEM (HT-7700, Hitachi).

### Nano-flow cytometry measurement (NFCM)

The NFCM analyzer (N30E nano-Analyzer, NanoFCM Inc., Xiamen, China) was utilized to assess the concentration and dimensions of EVs, following the manufacturer's prescribed protocol. To calibrate EV size and concentration, a 250 nm Silica Nanosphere Cocktail (NanoFCM Inc) was employed in the procedure. In summary, the EV samples, diluted to a 20% concentration in PBS, were incubated with FITC-labeled CD9 or FITC-labeled CD81 (BD Bioscience, USA) in a 3:2 ratio for 30 min at 37 °C. Subsequently, the stained EVs underwent two PBS washes, followed by centrifugation at 110,000 g at 4 °C for 1 h (CP100MX, Hitachi High-Technologies Corporation, Japan). The resulting EV pellet was resuspended in 50 μL PBS after discarding the supernatant. EV concentration, size, and surface proteins were assessed by recording events for 1 min. Subsequent data processing and plot generation were conducted using the nano-Analyzer software.

### Liquid chromatography with tandem mass spectrometry (LC–MS/MS) assay

An EASY-nLC 1200UHPLC system coupled with an Orbitrap Fusion Lumos (Thermo Fisher) was employed for all MS assays described in this study. Briefly, the digested peptides, resuspended in 0.1% formic acid supplemented with 2% acetonitrile, were separated using a 75 μm × 25 cm reversed-phase high-performance liquid chromatography (RP-HPLC) column packed with C18 beads (2 μm, Thermo Fisher). A gradient of acetonitrile ranging from 9 to 28% was utilized to elute the samples within 1.5 h, with a rapid increase to 80% acetonitrile accomplished within 20 min at a flow rate of 300 nL/min. Data acquisition was performed by the Orbitrap Fusion Lumos, employing alternating MS full-scans and MS2 scans at a spray voltage of 2.2 kV and an ion transfer capillary temperature of 300 °C. MS spectra collection utilized settings of 120000 resolution, 4 × 10^5^ automatic gain control, and a maximal injection time of 50 ms. Selected ions underwent fragmentation using a 3 s cycle by high collision dissociation normalized to 30% collision energy. Isolated windows were specified at 1.6 m/z, maintaining a resolution of 15000, and implementing a dynamic exclusion of 30 s. Ions unassigned or with a charge state of 1 + or > 7 +  were excluded from MS detection.

### Mass spectrometry (MS) data analysis

The MS data analysis was conducted as follows: Initially, the raw data underwent analysis using Proteome Discoverer (version 2.2). Subsequently, the processed data were aligned against the SwissProt human proteome database. The alignments were carried out with a 20 ppm precursor mass tolerance. Variable modifications included oxidation (Met) (+ 15.9949 Da) and acetylation (protein N-terminus) (+ 42.0106 Da), while carbamidomethylation (Cys) (+ 57.0215 Da) was set as a fixed modification. Peptides consisting of more than six amino acids were considered for analysis. Proteome Discoverer (PD) was employed to filter false discovery rates, utilizing a threshold of 1%. Identification of specific proteins relied on the presence of one or more peptides meeting these criteria. Whether the top 15 up- and down-regulated differentially expressed proteins between HC and BTI patients have been previously identified in exosomes from other conditions was explored via the ExoCarta database (http://www.exocarta.org/).

### Gene ontology (GO) annotation

GO analysis was conducted using the DAVID platform (version 6.8)on the liquid chromatography–mass spectrometry data of EVs obtained from both healthy controls and patients diagnosed with BTI^45^. To begin, identified protein IDs were converted to UniProt IDs for seamless mapping to GO IDs. This mapping was based on GO annotation proteome data sourced from the UniProt-GOA database (http://www.ebi.ac.uk/GOA/). The interProScan software was utilized to determine the functions of proteins not covered in the UniProt-GOA database. This determination was achieved through a protein sequence alignment method.Subsequently, the differentially expressed proteins were systematically categorized into three distinct classes: biological process, cellular component, and molecular function. This classification was carried out in accordance with the GO annotation database.

### Kyoto encyclopedia of genes and genomes pathway (KEGG) enrichment

KEGG analysis was conducted to delve into the underlying molecular interaction networks among the identified proteins. Differentially expressed proteins in EVs derived from both HC and patients diagnosed with BTI were annotated to the protein's KEGG database using the KEGG Automatic Annotation Server (KAAS) available through the KEGG online service tool.Following the annotation process, the results were further mapped to the KEGG pathway database utilizing the KEGG Mapper tool, also part of the KEGG online service. Finally, the enriched pathways were systematically categorized into hierarchical classes in accordance with the KEGG website's specified classifications.

### Western blot

EVs proteins were extracted using RIPA buffer on ice for 30 min. The protein concentration was determined using the BCA protein assay (Beyotime, China). To denature the proteins, the protein solution was mixed with 2 × Laemmli Sample Buffer and heated at 95 °C for 10 min. Subsequently, the denatured samples were loaded onto 12% SDS-PAGE gels, and the separated proteins were transferred to methanol-activated PVDF membranes. For detection of specific targets, 20 μg of total protein from each exosome derived from patients and healthy individuals was used. The PVDF membrane was blocked in 5% skim milk-TBST at room temperature for 1 h. Following this, the blocked membrane was incubated overnight at 4 °C with primary antibodies: CEACAM1 Rabbit mAb (1:1000, Abclonal, A11626), CRB3 Rat mAb (1:1000, Abcam, ab180835), CD9 Rabbit mAb (1:1000, Boster, BM4212), CD63 Rabbit mAb (1:1000, Abclonal, A19023), CD81 Rabbit mAb (1:1000, SAB, 41779), TSG101 Rabbit mAb (1:1000, Abcam, ab125011), and TSG101 Rabbit mAb (1:1000, Abclonal, A5789). These antibodies were diluted with 5% BSA. Following the primary antibody incubation, the membrane was further incubated with secondary antibodies: anti-Rabbit HRP-conjugated (1:10000, Invitrogen, 31460) and anti-Rat HRP-conjugated (1:10000, Invitrogen, 31460) respectively, in 5% skim milk-TBST for 1 h at room temperature. Protein signatures were captured using an imaging system (ChemiScope 3000 mini, Clinx Science Instruments, Shanghai, China). Image J software was used for quantifying western blot bands. Original blots/gels were presented in Supplementary Material 1.

### ELISA

Protein levels were determined using ELISA kits according to the manufacturer’s instructions: Human CEACAM1 ELISA Kit (EK1361, BOSTER, China) and Human Protein Crumbs Homolog 3 (CRB3) ELISA Kit (abx386665, abbexa, UK). A standard curve was generated, and the absorbance at 450 nm was evaluated to quantify protein levels.

### Statistical analysis

Statistical analysis was conducted on the datasets using the Student’s t-test, considering *P*-values less than 0.05 as statistically significant.

### Ethics approval and consent to participate

The study was approved by the Institutional Review Board of Xiamen Chang Gung Hospital (approval number: XMCGIRB2019031) and conducted in accordance with the guidelines of the Declaration of Helsinki. Informed consent was obtained from all participants before study.

### Supplementary Information

Supplementary Information 1.

Supplementary Information 2.

Supplementary Information 3.

## Data Availability

The datasets generated and/or analysed during the current study are available in the ProteomeXchange Consortium (http://proteomecentral.proteomexchange.org) via the iProX partner repository with the dataset identifier PXD045960.
