# Supplementary material for: Proteomic analysis of serum extracellular vesicles from biliary tract infection patients to identify novel biomarkers
Source: Sci Rep. 2024 Mar 8;14:5707. doi: 10.1038/s41598-024-56036-y (PMC10923810; doi:10.1038/s41598-024-56036-y)
Supplement: Supplementary file 1 — Supplementary Information 1. [file 41598_2024_56036_MOESM1_ESM.pdf]

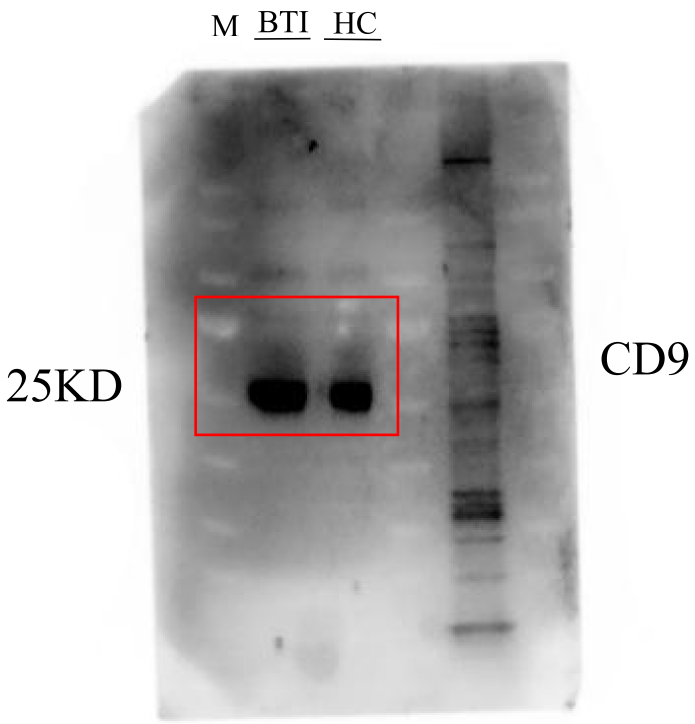

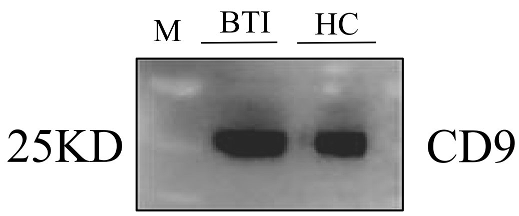

CD9 Supplementary Gel for Figure 1E

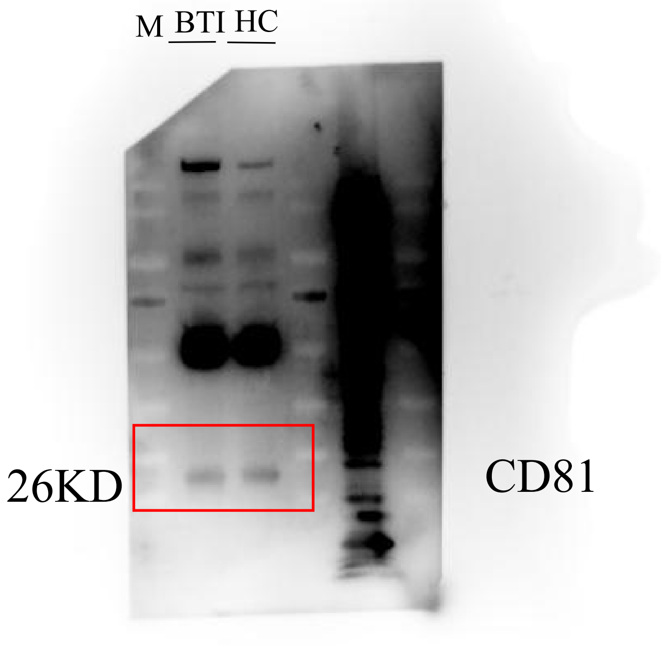

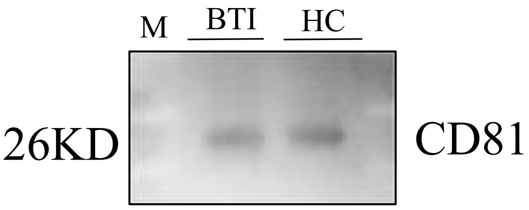

CD81 Supplementary Gel for Figure 1E

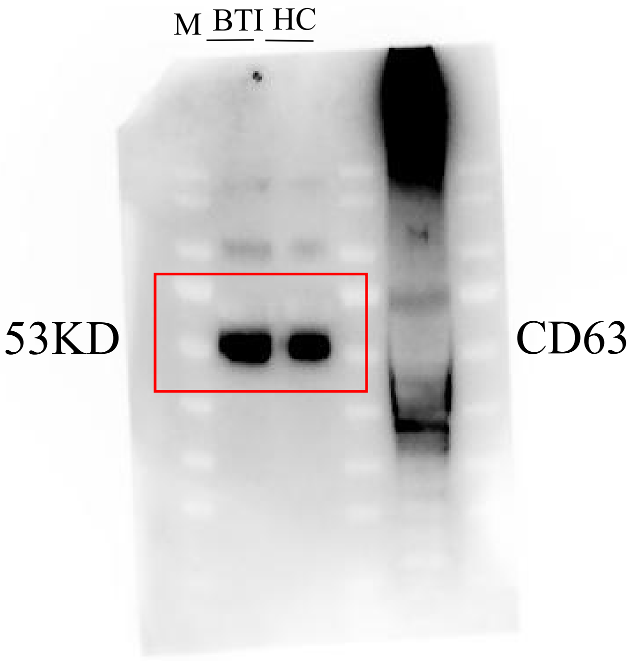

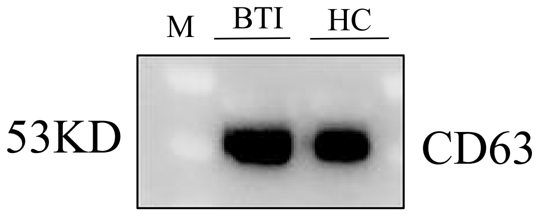

CD63 Supplementary Gel for Figure 1E

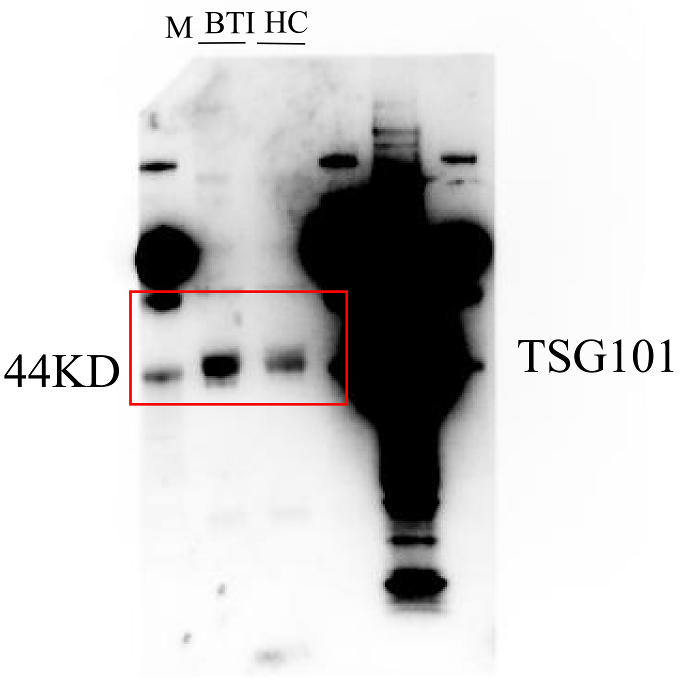

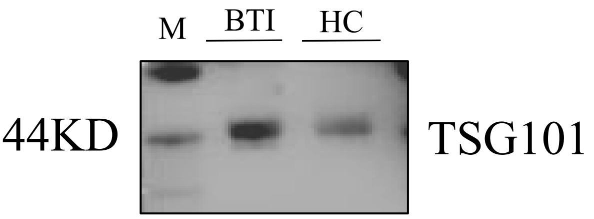

TSG101 Supplementary Gel for Figure 1E

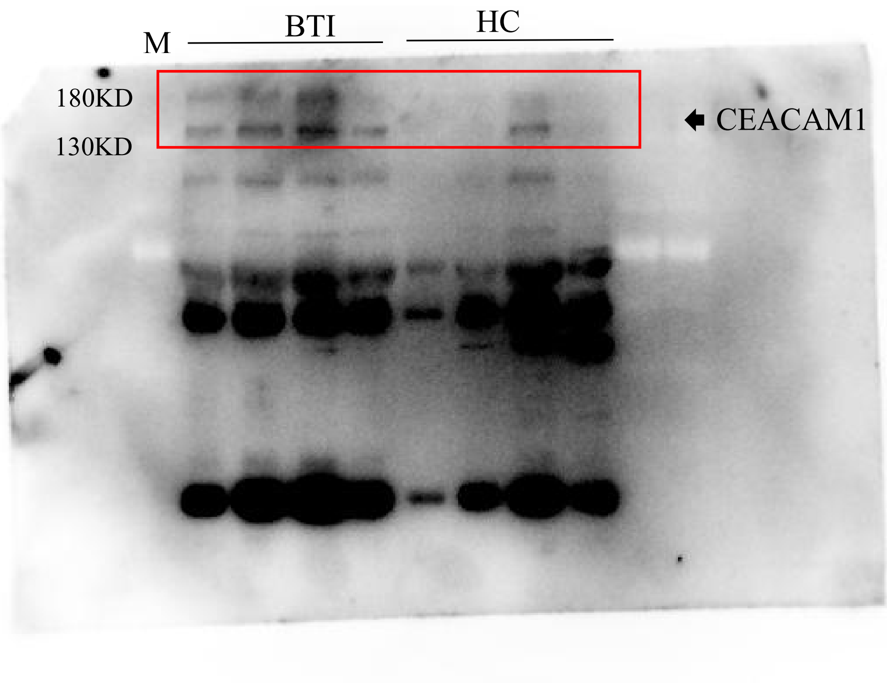

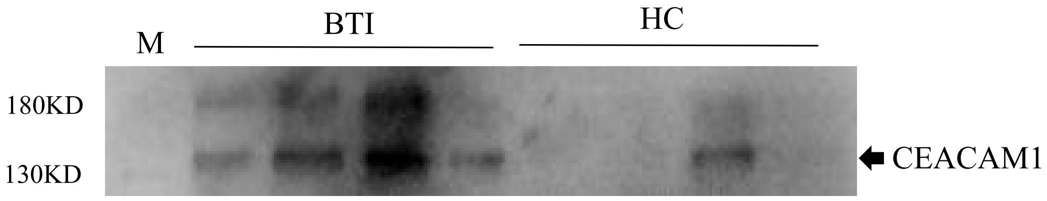

CEACAM1 Supplementary Gel for Figure 5A

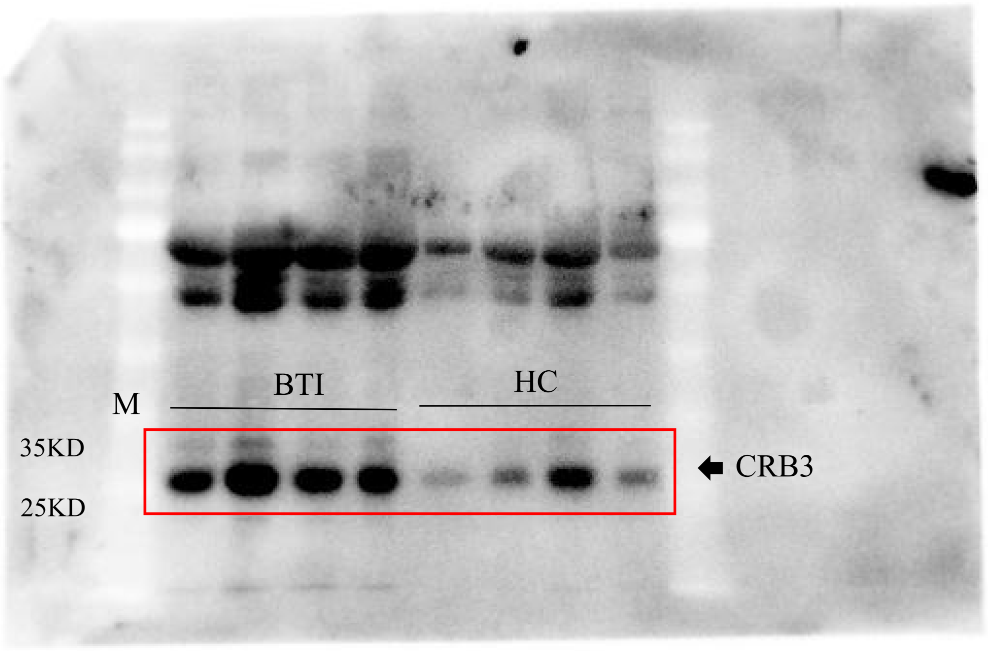

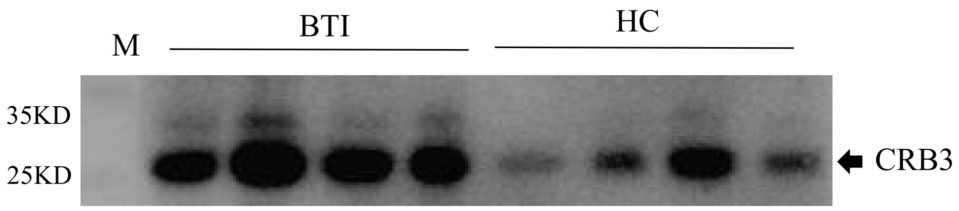

CRB3 Supplementary Gel for Figure 5A

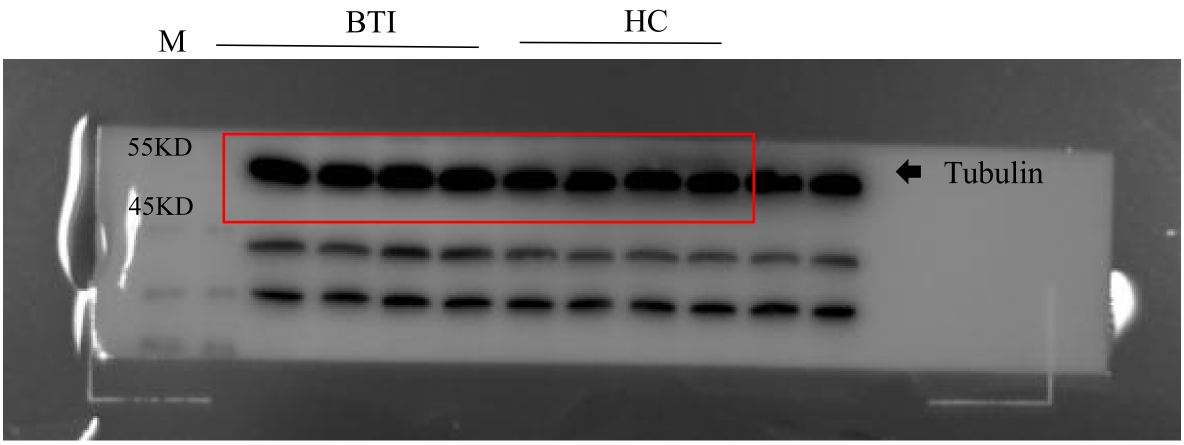

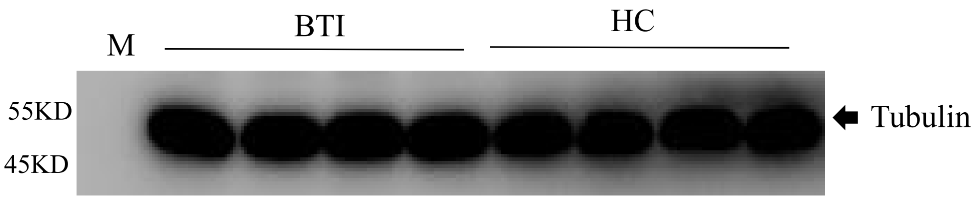

Tubulin Supplementary Gel for Figure 5A
